# Dietary zinc intake, serum zinc concentrations, and disease activity in children with juvenile idiopathic arthritis: a case-control study

**DOI:** 10.3389/fnut.2026.1940130

**Published:** 2026-09-11

**Authors:** Oksana Boyarchuk, Nataliia Kovalchuk

**Affiliations:** Department of Children's Diseases and Pediatric Surgery, I.Horbachevsky Ternopil National Medical University, Ternopil, Ukraine

**Keywords:** children, dietary intake, disease activity, JADAS-27, juvenile idiopathic arthritis, nutrition, serum zinc, zinc

## Abstract

**Introduction:**

Zinc is an essential trace element involved in immune regulation and inflammatory responses. Children with juvenile idiopathic arthritis (JIA) may be at increased risk of nutritional deficiencies due to chronic inflammation, altered dietary intake, and long-term treatment. However, evidence regarding dietary zinc intake and serum zinc status in JIA remains inconsistent. This study aimed to evaluate dietary zinc intake and serum zinc concentrations in children with JIA and healthy controls and to explore their potential relationship with disease activity.

**Methods:**

This unmatched case-control study included children with JIA and healthy controls aged 6–18 years. Dietary zinc intake was assessed using a semi-quantitative food frequency questionnaire. Serum zinc concentrations were measured using a colorimetric assay. Demographic, clinical, laboratory, dietary, and treatment characteristics were analyzed according to serum zinc status within the JIA cohort.

**Results:**

Children with JIA had significantly lower dietary zinc intake than healthy controls (median 5.85 vs. 7.59 mg/day, *p* = 0.0019). Dietary intake was below the age- and sex-specific recommended level in 96.4% of children with JIA compared with 62.1% of controls (*p* < 0.0001). Mean serum zinc concentrations did not differ significantly between groups (*p* = 0.8872). Low serum zinc concentration (<11 μmol/L) was more frequent in children with JIA than controls (29.8% vs. 14.5%), although the difference was not statistically significant (OR 2.50, 95% CI 0.97–6.42; *p* = 0.0572). Within the JIA cohort, low serum zinc concentration was associated with higher erythrocyte sedimentation rate, patient and physician global assessment scores, and higher clinical JADAS-27 scores. However, overall JADAS-27 did not differ significantly between groups, and serum zinc concentration was not significantly correlated with JADAS-27 or clinical JADAS-27 when analyzed as a continuous variable.

**Conclusions:**

Children with JIA had substantially lower dietary zinc intake than healthy controls, whereas mean serum zinc concentrations did not differ significantly between the groups. Low serum zinc concentration was associated with selected measures of disease activity but did not show an independent or causal relationship with systemic inflammation. Prospective studies are needed to clarify the temporal relationships between dietary zinc intake, serum zinc status, inflammation, and disease activity and to determine whether nutritional interventions have clinically relevant effects.

## Introduction

Juvenile idiopathic arthritis (JIA) is the most common chronic inflammatory joint disease in childhood and currently comprises seven clinically heterogeneous categories with distinct clinical and laboratory characteristics (1, 2). Despite substantial advances in treatment, JIA remains an important cause of both short- and long-term functional disability in a considerable proportion of affected children (3, 4).

The epidemiology of JIA varies across geographic regions, ethnic populations, and classification approach, making direct comparisons between studies challenging and highlighting the need for local investigations of clinical characteristics and potentially modifiable factors influencing disease course (3, 5). Worldwide, JIA affects approximately 3 million children and young people (5). In Germany, the reported prevalence ranges from 133 to 168 per 100,000 children, with an incidence of 34–60 per 100,000 population (3).

Among the potentially modifiable factors influencing disease outcomes, nutritional status has attracted increasing attention. Children with JIA are at increased risk of developing nutritional and metabolic disturbances due to chronic inflammation, reduced physical activity, altered dietary intake, and long-term pharmacological treatment (6, 7). Nutritional disturbances may contribute to impaired growth and other adverse health outcomes, while treatment-related gastrointestinal symptoms may further influence dietary intake.

Zinc is an essential trace element that plays a critical role in growth and development, gene transcription, intracellular signaling, and the proper functioning of both innate and adaptive immunity (8–10). Disruption of zinc homeostasis impairs the survival, proliferation, and differentiation of immune cells, particularly T-lymphocyte subsets, whereas chronic zinc deficiency has been associated with an enhanced pro-inflammatory immune response (11, 12). In addition, zinc is involved in the regulation of nuclear factor kappa B (NF-κB) signaling, oxidative stress, and cytokine production, providing a biological basis for investigating its potential relationship with inflammatory processes (9, 13). Alterations in zinc status have been reported in some inflammatory conditions, including arthritis, although their clinical significance and relationship to disease activity remain unclear (10).

Evidence regarding zinc status in children with JIA suggests that both inadequate dietary intake and altered serum zinc concentrations may occur; however, published findings remain inconsistent. Early studies demonstrated lower serum zinc concentrations in children with juvenile arthritis compared with healthy controls, accompanied by dietary zinc intake below recommended levels (14). Similarly, among children with polyarticular juvenile chronic arthritis, reduced serum zinc concentrations were associated with higher erythrocyte sedimentation rate (ESR), supporting the influence of inflammatory activity on zinc metabolism (15).

In contrast, a Brazilian study reported dietary zinc intake below recommended values in most patients, while serum zinc concentrations did not differ significantly from those of healthy controls and were not associated with disease characteristics (16). More recent evidence remains conflicting. A 2024 study found significantly lower mean serum zinc concentrations in patients with JIA and demonstrated a negative correlation between zinc levels and disease duration (17). Conversely, another recent study reported no significant difference in serum zinc concentrations between children with JIA and healthy controls (18).

Overall, suboptimal dietary zinc intake has been described in children with JIA (5). Reduced serum zinc concentrations have also been reported in several studies, particularly among patients with polyarticular disease (6, 13, 14). However, the relationship between zinc status and disease activity, severity, or duration remains inconclusive (17, 19).

It should be noted that interpretation of serum zinc concentrations in children with JIA requires caution because serum zinc reflects not only dietary zinc status but also the systemic inflammatory response. Plasma or serum zinc is a commonly used biomarker for assessing zinc status at the population level; however, its concentration decreases during the acute-phase response owing to the redistribution of zinc from the circulation to the liver (8). Furthermore, inflammation may reduce circulating zinc concentrations through decreased albumin levels, as albumin is a negative acute-phase protein and the principal carrier of zinc in plasma (20). Serum zinc concentrations may also vary according to age, sex, time of blood sampling, and fasting status, which should be considered when interpreting individual measurements. Consequently, reduced serum zinc concentrations in children with active arthritis do not necessarily indicate nutritional zinc deficiency and should be interpreted in conjunction with dietary assessment, clinical findings, and inflammatory markers (8).

Taken together, the assessment of both dietary zinc intake and serum zinc concentrations in children with JIA is clinically relevant because it integrates nutritional, immunological, and inflammatory aspects of the disease. Given the inconsistency of the available evidence and the methodological challenges associated with interpreting serum zinc as a biomarker of zinc status, further studies are warranted to clarify zinc status in children with JIA and to determine its potential relationship with disease activity and clinical outcomes. The aim of our study was to evaluate dietary zinc intake and serum zinc concentrations in children with JIA and healthy controls and to explore their potential relationship with disease activity.

## Materials and methods

### Study design and participants

This case-control study included children diagnosed with JIA and apparently healthy children aged 6–18 years. Children with JIA were recruited during hospitalization or routine outpatient follow-up visits at the Regional Children's Hospital, whereas healthy controls were enrolled during scheduled preventive health examinations.

Patients were eligible for inclusion in the JIA group if they fulfilled the International League of Associations for Rheumatology (ILAR) classification criteria for JIA (2) and provided written informed consent from the child and/or their parents or legal guardians. Children with acute infectious diseases, other chronic inflammatory or systemic diseases, or renal dysfunction were excluded. The control group consisted of apparently healthy children without acute or chronic illnesses. Written informed consent was obtained from the parents or legal guardians, with child assent obtained when appropriate.

### Clinical assessment

Demographic information, including age, sex, place of residence, and parental educational level, was collected through structured interviews. Anthropometric data, including body mass index (BMI), were also recorded. Participants and/or their parents were asked about the use of zinc-containing dietary supplements, and none of the children included in the study reported zinc supplementation. Medical records were reviewed to obtain clinical information, including JIA category, disease duration, disease activity, laboratory findings, and current treatment, including methotrexate, glucocorticoids, and biological therapy. Anemia was defined according to age-specific World Health Organization (WHO) hemoglobin criteria, and thrombocytopenia was defined as a platelet count < 150 × 10^9^/L.

Disease activity was assessed using the Juvenile Arthritis Disease Activity Score based on 27 joints (JADAS-27). The JADAS-27 includes four components: physician's global assessment of disease activity measured on a 10-cm visual analog scale (VAS); parent/patient global assessment of wellbeing measured on a 10-cm VAS (parent assessment was used for children ≤ 9 years of age); active joint count (0–27 joints); and an acute-phase reactant. In the present study, ESR was used as the acute-phase reactant component of the JADAS-27. ESR was normalized to a 0–10 scale using the formula [ESR (mm/h) – 20]/10, with values below zero assigned a value of 0 and values above 10 capped at 10. The resulting JADAS-27 score ranged from 0 to 57. Clinical JADAS-27 was calculated using the three clinical components of the JADAS-27, without the acute-phase reactant. CRP was analyzed separately as an inflammatory marker and was not included in the calculation of JADAS-27.

Active disease was defined according to established JADAS-27 disease activity criteria (21).

### Dietary assessment

Dietary zinc intake was assessed using a semi-quantitative food frequency questionnaire (FFQ) covering the major zinc-containing foods commonly consumed by children. The questionnaire was pilot-tested in children comparable in age to the study population before implementation in the main study to assess the clarity and feasibility of the questions and the comprehensiveness of the listed food items.

Participants, with assistance from their parents when necessary, reported their usual intake of the listed food items during the previous week, including the frequency of consumption and estimated portion size. Standard portion sizes and household measures familiar to families were used to improve consistency in portion-size estimation. For younger participants, parental assistance was required to improve the accuracy of dietary recall.

The reported intake during the preceding week was converted into an estimated average daily intake, and daily zinc intake was calculated using a food composition database containing zinc concentrations in individual food products (22). Estimated daily zinc intake was subsequently compared with age- and sex-specific recommended dietary intake levels established by the Ministry of Health of Ukraine (Order No. 1073, September 3, 2017) and international dietary reference values for children and adolescents (23).

According to these national standards, the recommended daily zinc intake is 10 mg/day for children aged 6–10 years, 15 mg/day for boys and 12 mg/day for girls aged 11–13 years, and 15 mg/day for boys and 13 mg/day for girls aged 14–17 years. Reported dietary zinc intake below the applicable age- and sex-specific recommended level was classified as “below the recommended intake level” and was not interpreted as evidence of individual zinc deficiency.

Information on the use of zinc-containing dietary supplements was also collected as part of the questionnaire. None of the participants reported the use of zinc-containing supplements.

Total energy intake was not used to energy-adjust dietary zinc intake in the primary analysis; therefore, the reported values represent absolute estimated daily zinc intake rather than energy-adjuste.

### Laboratory assessment

Venous blood samples were collected under standard clinical conditions. Serum zinc concentration was measured using a colorimetric assay (Zinc Assay Kit, Elabscience, E-BC-K137-M, USA) according to the manufacturer's instructions. Absorbance was determined using a Multiskan FC microplate photometer (Thermo Fisher Scientific, Finland). A serum zinc concentration of 11.0 μmol/L represented the lower reference limit specified by the manufacturer for the assay. Accordingly, serum zinc concentrations were categorized as < 11.0 μmol/L or ≥11.0 μmol/L for the categorical analyses. This threshold was used as an assay-specific analytical reference criterion and was not interpreted as a definitive marker of nutritional zinc deficiency.

Routine inflammatory markers, including ESR and CRP, were obtained from the clinical laboratory records. These markers were analyzed separately in relation to serum zinc concentrations. ESR was used as the acute-phase reactant component of the JADAS-27, whereas CRP was not included in the JADAS-27 calculation.

### Relationship to previous study

A proportion of participants in the present study had previously been included in our study of dietary and serum magnesium status in children with JIA (6). Some dietary questionnaire data, clinical data, and stored blood samples therefore overlap between the two studies. Of the 47 children with JIA and available serum zinc measurements and 56 children with available dietary zinc intake data in the present study, 34 and 26, respectively, had participated in the previous study. In addition, 62 healthy controls included in the present study had also participated in the previous study. The present study addresses a distinct research question, focusing on zinc status and its relationship with disease activity, and includes a substantially larger cohort and additional analyses not reported in the previous study.

### Ethical considerations

The study was conducted in accordance with the ethical principles of the Declaration of Helsinki (1964, revised in 2013). The study protocol was approved by the Bioethics Committee of I. Horbachevsky Ternopil National Medical University (Protocol No. 60, September 1, 2020). Written informed consent was obtained from all participants and/or their parents or legal guardians before enrollment.

### Statistical analysis

Statistical analyses were performed using STATISTICA version 10.0 (StatSoft Inc., Tulsa, OK, USA) and Microsoft Excel 2003 (Microsoft Corporation, Redmond, WA, USA).

For continuous variables, distributional characteristics were assessed before analysis. Normally distributed variables are presented as mean ± standard deviation (SD) and were compared between groups using the independent-samples *t*-test; Welch's *t*-test was used when equality of variances could not be assumed. Non-normally distributed variables are presented as median [interquartile range (IQR)] and were compared using the Mann–Whitney U test. Categorical variables are presented as *n* (%) and were compared using Fisher's exact test. Mutually exclusive JIA categories were analyzed as a single categorical variable using an overall omnibus test.

For the comparison of serum zinc status between children with JIA and healthy controls, the odds ratio (OR) with 95% confidence interval (CI) was calculated for serum zinc concentrations < 11.0 μmol/L. Spearman's rank correlation coefficient (ρ) was used to assess associations between serum zinc concentrations or dietary zinc intake and continuous measures of disease activity, inflammatory markers, and disease duration.

Laboratory and clinical variables were analyzed based on available data, and denominators were reported for variables with missing observations. Analyses of clinical characteristics according to serum zinc status were considered exploratory. No adjustment for multiple comparisons was applied, and all tests were two-sided, with a *p* value < 0.05 considered statistically significant.

## Results

### Baseline characteristics of the study population

The study included 56 children with JIA and 66 healthy controls. The demographic characteristics of the study groups are summarized in Table 1. The proportions of boys and girls did not differ significantly between children with JIA and healthy controls [28/56 (50.0%) vs. 36/66 (54.5%], respectively; *p* = 0.7164). Mean age did not differ significantly between the groups (12.14 ± 3.62 vs. 11.09 ± 3.37 years, respectively; *p* = 0.100). Similarly, BMI did not differ significantly between children with JIA and healthy controls (18.21 ± 3.75 vs. 19.65 ± 4.09 kg/m^2^, respectively; *p* = 0.0567).

**Table 1:** Baseline characteristics of patients with JIA and healthy controls.

Characteristic	JIA	Healthy controls	*p-*value
	*n* = 56	*n* = 66	
Male, *n* (%)	28 (50.0)	36 (54.5)	0.7164
Female, *n* (%)	28 (50.0)	30 (45.5)	
Place of residence: rural, *n* (%)	29 (51.8)	18 (27.3)	**0.0087**
Urban, *n* (%)	27 (48.2)	48 (72.7)	
Parent's education: higher, *n* (%)	19 (33.9)	34 (51.5)	0.0669
Secondary, *n* (%)	37 (66.1)	32 (48.4)	
Age at visit, years (mean ± SD)	12.14 ± 3.62	11.09 ± 3.37	0.100
BMI, kg/m^2^ (mean ± SD, min-max)	18.21 ± 3.75 (12.62–27.72)	19.65 ± 4.09 (13.17–28.26)	0.0567

SD, standard deviation; statistically significant values are highlighted in bold.

Children with JIA were significantly more likely to live in rural areas than healthy controls [29/56 (51.8%) vs. 18/66 (27.3%), respectively; *p* = 0.0087]. A higher proportion of parents in the control group had completed higher education compared with parents of children with JIA [34/66 (51.5%) vs. 19/56 (33.9%), respectively], but the difference was not statistically significant (*p* = 0.0669).

None of the participants in either group reported the use of zinc-containing dietary supplements. Thus, zinc supplementation was not identified as a potential source of between-group differences in serum zinc concentrations.

### Clinical characteristics of children with JIA

**Figure 1:**
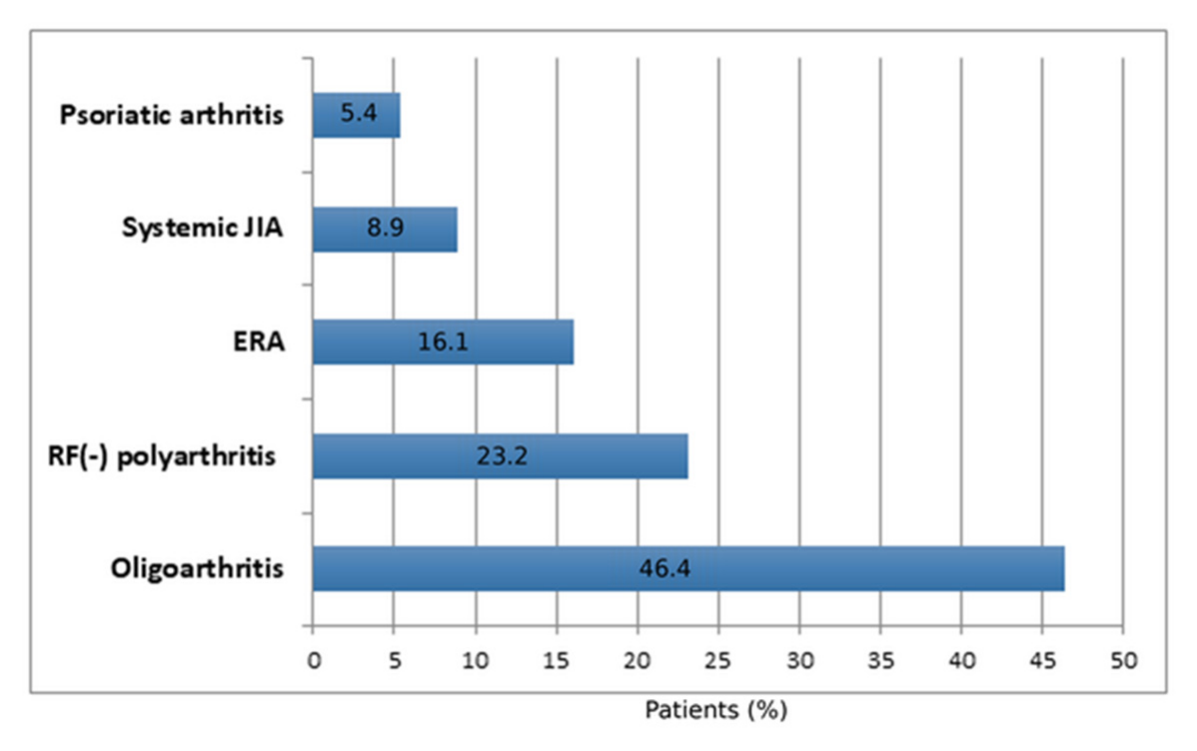
Distribution of JIA subtypes among the study participants (*n* = 56). Oligoarthritis was the predominant subtype [26/56 (46.4%)], followed by RF-negative polyarthritis [13/56 (23.2%)], enthesitis-related arthritis [9/56 (16.1%)], systemic JIA [5/56 (8.9%)], and psoriatic arthritis [3/56 (5.4%)]. Percentages are based on the total number of patients with JIA (*n* = 56).

The mean disease duration was 3.74 ± 2.89 years (range, 4 months−13 years). Oligoarthritis was the most common JIA subtype, accounting for 26/56 (46.4%) patients, followed by RF-negative polyarthritis [13/56 (23.2%)], enthesitis-related arthritis [9/56 (16.1%)], systemic JIA [5/56 (8.9%)], and psoriatic arthritis [3/56 (5.4%)] (Figure 1). No patients with RF-positive polyarthritis or undifferentiated arthritis were identified.

Uveitis was diagnosed in 4/56 (7.1%) children, including three patients with oligoarthritis and one with enthesitis-related arthritis. ANA positivity was observed in 29/54 (53.7%) patients with available ANA results and was most frequent among children with oligoarthritis. HLA-B27 was positive in 12/54 (22.2%) patients with available results, predominantly among those with enthesitis-related arthritis. Rheumatoid factor was negative in all 56 patients.

At the time of assessment, active disease was present in 30/56 (53.6%) children. The median JADAS-27 score was 3.0 (IQR, 0–6; range, 0–23).

Methotrexate was the most commonly prescribed disease-modifying antirheumatic drug, used by 48/56 (85.7%) patients. Glucocorticoids were administered to 19/56 (33.9%) children, including 11 who received systemic glucocorticoids (short courses during disease flares or maintenance therapy) and 8 who underwent intra-articular glucocorticoid injections.

Biological therapy was administered to 29/56 (51.8%) patients. Adalimumab was used in 19/56 (33.9%) patients, while tocilizumab was administered to 10/56 (17.9%) patients.

### Dietary zinc intake

Dietary zinc intake is presented in Table 2. Children with JIA had significantly lower daily dietary zinc intake than healthy controls, with a median intake of 5.85 mg/day (IQR, 4.59–7.53) compared with 7.59 mg/day (IQR, 5.84–11.26) in healthy controls (*p* = 0.0019). Dietary zinc intake was below the age- and sex-specific recommended level in 54/56 (96.4%) children with JIA compared with 41/66 (62.1%) healthy controls (*p* < 0.0001).

**Table 2:** Daily dietary zinc intake in patients with JIA and healthy controls.

Group	*n*	Daily dietary zinc intake, mg/day median [IQR]	Dietary zinc intake below the recommended level, *n* (%)
JIA	56	5.85 [4.59–7.53]	54/56 (96.4)
Healthy controls	66	7.59 [5.84–11.26]	41/66 (62.1)
*p-*valu*e*		**0.0019**	**< 0.0001**

IQR, interquartile range; statistically significant values are highlighted in bold.

### Serum zinc concentrations

Serum zinc concentrations were available for 47/56 (83.9%) children with JIA and 62/66 (93.9%) healthy controls (Table 3). Mean serum zinc concentrations did not differ significantly between children with JIA and healthy controls (13.66 ± 4.32 vs. 13.76 ± 3.02 μmol/L, respectively; *p* = 0.8872).

**Table 3:** Serum zinc concentrations in patients with JIA and healthy controls.

Group	Serum zinc measurements, *n*/*N*	Serum zinc concentrations, μmol/L (mean±SD)	Serum zinc < 11.0 μmol/L, *n* (%)
JIA	47/56	13.66 ± 4.32	14 (29.8)
Healthy controls	62/66	13.76 ± 3.02	9 (14.5)
*p-*value		0.8872	**0.0615**

SD, standard deviation; statistically significant values are highlighted in bold. Categorical variables were compared using Fisher's exact test.

Serum zinc concentrations below 11.0 μmol/L were observed in 14/47 (29.8%) children with JIA compared with 9/62 (14.5%) healthy controls. Although the proportion of children with low serum zinc concentrations was higher among those with JIA, the difference was not statistically significant according to Fisher's exact test (29.8% vs. 14.5%, *p* = 0.0615). Logistic regression analysis yielded an odds ratio of 2.4983 (95% CI, 0.9725–6.4179; *p* = 0.0572).

### Clinical characteristics according to serum zinc status

Children with JIA were stratified according to serum zinc concentration using a predefined threshold of 11.0 μmol/L (Table 4). No significant differences were observed between children with serum zinc concentrations below and above this threshold with respect to sex, place of residence, age, BMI, disease duration, JIA subtype, uveitis, ANA or HLA-B27 positivity, or treatment with methotrexate, glucocorticoids, or biological agents (all *p* > 0.05). Similarly, dietary zinc intake did not differ significantly between the two groups [median, 6.15 (IQR, 4.86–7.15) vs. 5.58 (IQR, 4.39–7.38) mg/day; *p* = 0.6170].

**Table 4:** Comparison of clinical characteristics of JIA according to zinc status.

Characteristic	Serum zinc < 11.0 μmol/L	Serum zinc ≥11.0 μmol/	*p-*value
	*n* = 14	*n* = 33	
	*n* (%)	*n* (%)	
Male	5/14 (35.7)	20/33 (60.6)	0.2006
Female	9/14 (64.3)	13/33 (39.4)	
Place of residence: rural	8/14 (57.1)	16/33 (48.5)	0.7516
Urban	6/14 (42.9)	17/33 (51.5)	
Age at visit, years, median [IQR]	11.00 [9.00–14.50]	12.00 [10.00–17.00]	0.1526
BMI, kg/m^2^, median [IQR]	16.09 [14.54–19.84]	17.78 [15.83–21.16]	0.4177
JIA duration, years, median [IQR]	2.25 [2.00–3.75]	3.00 [2.00–7.00]	0.3023
JIA category, overall			0.5867
Oligoarthritis	5/14 (35.7)	14/33 (42.4)	
Polyarthritis	3/14 (21.4)	9/33 (27.3)	
ERA	2/14 (14.3)	6/33 (18.2)	
Systemic JIA	2/14 (14.3)	3/33 (9.1)	
Psoriatic arthritis	2/14 (14.3)	1/33 (3.0)	
Uveitis	1/14 (7.1)	2/33 (6.1)	1.000
ANA +	5/13 (38.5)	19/32 (59.4)	0.3234
HLA-B27 +	5/14 (35.7)	6/31 (19.4)	0.2771
Active disease	10/14 (71.4)	15/33 (45.5)	0.1232
CRP, mg/L, median [IQR]	1.78 [0.35–12.05] (*n* = 12)	0.42 [0.23–0.86] (*n* = 30)	0.0620
ESR, mm/h, median [IQR]	9.00 [4.00–27.50]	4.00 [4.00–5.00]	**0.0170**
Active joints, *n*, median [IQR]	1.00 [0–2.00]	0 [0–1.00]	0.0695
Patient global assessment (VAS), median [IQR]	3.00 [1.00–4.00]	1.00 [0–2.00]	**0.0211**
Physician global assessment (VAS), median [IQR]	2.50 [0.25–3.88]	1.00 [0–1.00]	**0.0222**
JADAS-27, median [IQR]	7.90 [2.56–13.38]	2.40 [0.70–4.40]	**0.0560**
Clinical JADAS-27, median [IQR]	7.00 [2.00–10.38]	2.00 [0–4.00]	**0.0310**
Glucocorticosteroid therapy, overall	6/14 (42.9)	10/33 (30.3)	0.4929
Systemic	5/14 (35.7)	6/33 (18.2)	
Intraarticular	1/14 (7.1)	4/33 (12.1)	
Methotrexate	12/14 (85.7%)	28/33 (84.8%)	1.000
Biologic therapy, overall	7/14(50.0)	20/33 (60.6)	0.5354
Adalimumab	5/14 (35.7)	12/33 (36.4)	
Tocilizumab	2/14 (14.3)	8/33 (24.2)	
Anemia	3/14 (21.4)	2/33 (6.1)	0.1481
Thrombocytopenia	0/14 (0.0)	2/32 (6.3)	1.000
Dietary zinc intake, mg/day, median [IQR]	6.15 [4.86–7.15]	5.58 [4.39–7.38]	0.6170

JIA, juvenile idippathic arthritis; ERA, enthesitis-related arthritis; ANA, antinuclear antibodies; HLA-B27, human leukocyte antigen B27; CRP, C-reactive protein; ESR, erythrocyte sedimentation rate; VAS, visual analog scale; IQR, interquartile range; JADAS-27, Juvenile Arthritis Disease Activity Score based on 27 joints; For JADAS-27 calculation, normalized value of ESR was used as the acute-phase reactant component. Clinical JADAS-27 was calculated without ESR value. Statistically significant values are highlighted in bold. Denominators indicate the number of participants with available data.

Several measures of disease activity differed between the groups. ESR was higher in children with serum zinc concentrations below 11.0 μmol/L than in those with concentrations ≥11.0 μmol/L [median, 9.00 (IQR, 4.00–27.50) vs. 4.00 (IQR, 4.00–5.00) mm/h; *p* = 0.0170]. Patient Global Assessment scores were also higher in the low-serum-zinc group [median, 3.00 (IQR, 1.00–4.00) vs. 1.00 (IQR, 0–2.00); *p* = 0.0211], as were Physician Global Assessment scores [median, 2.50 (IQR, 0.25–3.88) vs. 1.00 (IQR, 0–1.00); *p* = 0.0222]. Clinical JADAS-27 was significantly higher among children with serum zinc concentrations below 11.0 μmol/L [median, 7.00 (IQR, 2.00–10.38) vs. 2.00 (IQR, 0–4.00); *p* = 0.0310].

In contrast, the difference in JADAS-27 did not reach statistical significance [median, 7.90 (IQR, 2.56–13.38) vs. 2.40 (IQR, 0.70–4.40); *p* = 0.0560]. Similarly, the number of active joints was higher in the low-serum-zinc group but did not differ significantly between the groups [median, 1.00 (IQR, 0–2.00) vs. 0 (IQR, 0–1.00); *p* = 0.0695]. CRP concentrations also did not differ significantly between the groups [median, 1.78 (IQR, 0.35–12.05) vs. 0.42 (IQR, 0.23–0.86) mg/L; *p* = 0.0620]. Although active disease was more frequent among children with serum zinc concentrations below 11.0 μmol/L [10/14 (71.4%) vs. 15/33 (45.5%)], the difference was not statistically significant (*p* = 0.1232).

To further assess the relationship between zinc status and disease activity, Spearman rank correlation analyses were performed using serum zinc concentrations and dietary zinc intake as continuous variables (Table 5). Serum zinc concentrations showed weak inverse correlations with JADAS-27 (ρ = −0.239, *p* = 0.106), Clinical JADAS-27 (ρ = −0.255, *p* = 0.084), ESR (ρ = −0.210, *p* = 0.156), CRP (ρ = −0.197, *p* = 0.210), and active joint count (ρ = −0.234, *p* = 0.114); none of these correlations reached statistical significance. A weak inverse correlation was observed between serum zinc concentration and Physician Global Assessment (ρ = −0.293, *p* = 0.046).

**Table 5:** Correlations of serum zinc concentrations and dietary zinc intake with disease activity and inflammatory markers in children with JIA.

Variable	Serum zinc, ρ	*p-*value	Dietary zinc intake, ρ	*p-*value
JADAS-27	−0.239	0.106	−0.146	0.327
Clinical JADAS-27	−0.255	0.084	−0.146	0.327
ESR	−0.210	0.156	−0.117	0.433
CRP	−0.197	0.210	−0.133	0.401
Active joint count	−0.234	0.114	−0.135	0.366
Patient/parent global assessment	−0.269	0.067	−0.121	0.419
Physician global assessment	−0.293	0.046	−0.160	0.282
Disease duration	0.093	0.534	0.291	0.047
Dietary zinc intake	−0.132	0.376	—	—

JADAS-27, Juvenile Arthritis Disease Activity Score based on 27 joints; Clinical JADAS-27 was calculated without ESR value; ESR, erythrocyte sedimentation rate; CRP, C-reactive protein. Spearman's rank correlation coefficient (ρ) was used. All tests were two-sided.

Dietary zinc intake was not significantly correlated with serum zinc concentration (ρ = −0.132, *p* = 0.376), JADAS-27 (ρ = −0.146, *p* = 0.327), Clinical JADAS-27 (ρ = −0.146, *p* = 0.327), ESR (ρ = −0.117, *p* = 0.433), CRP (ρ = −0.133, *p* = 0.401), active joint count (ρ = −0.135, *p* = 0.366), patient/parent global assessment (ρ = −0.121, *p* = 0.419), or Physician Global Assessment (ρ = −0.160, *p* = 0.282). A weak positive correlation was observed between dietary zinc intake and disease duration (ρ = 0.291, *p* = 0.047).

## Discussion

In the present study, we evaluated both dietary zinc intake and serum zinc concentrations in children with JIA and examined their relationships with clinical disease activity. Three main findings emerged. First, children with JIA had significantly lower dietary zinc intake than healthy controls, and 96.4% of patients had an estimated intake below the age- and sex-specific Ukrainian recommended level. Second, mean serum zinc concentrations did not differ significantly between children with JIA and healthy controls, although serum zinc concentrations below 11.0 μmol/L were more frequent among children with JIA than controls (29.8% vs. 14.5%), without reaching statistical significance. Third, within the JIA cohort, several indicators of disease activity differed according to serum zinc status: children with serum zinc concentrations < 11.0 μmol/L had higher ESR, patient and physician global assessments, and clinical JADAS-27 scores than those with concentrations ≥11.0 μmol/L. However, the difference in overall JADAS-27 did not reach statistical significance, and serum zinc concentrations analyzed as a continuous variable were not significantly correlated with JADAS-27 or clinical JADAS-27. These findings suggest that low serum zinc concentrations may be associated with selected measures of disease activity, but they do not establish an independent or continuous relationship between serum zinc concentration and disease activity.

To our knowledge, this is one of the few studies from Eastern Europe to simultaneously evaluate dietary zinc intake, serum zinc concentrations, and their relationship with disease activity in children with JIA.

Our finding of substantially lower dietary zinc intake among children with JIA is consistent with evidence that dietary intake below recommended levels may occur in pediatric rheumatic diseases (6, 16, 24). Several factors may contribute to dietary zinc intake below the recommended level in children with chronic inflammatory disease. Factors that may directly reduce food intake include impaired appetite, altered dietary habits, gastrointestinal symptoms, and treatment-related changes in appetite or food preferences (6). In contrast, chronic inflammation and disease activity may influence nutrient metabolism and requirements, while growth, body composition, and overall nutritional status may affect the relationship between dietary intake and circulating nutrient concentrations. Reduced physical activity and treatment-related changes in body composition may also influence nutritional status without necessarily directly reducing zinc intake. A recent systematic review and meta-analysis similarly highlighted the occurrence of dietary intake below recommended levels among children and adolescents with JIA and emphasized the importance of nutritional assessment as part of comprehensive disease management (24).

Importantly, more than half of the healthy controls in our study also had dietary zinc intake below the applicable national recommendation (62.1%), suggesting that dietary zinc intake below recommended levels may represent a broader nutritional concern among Ukrainian children rather than a finding restricted to JIA. Nevertheless, the prevalence was substantially higher among children with JIA. This difference should be interpreted in the context of the sociodemographic imbalance between the study groups. Children with JIA were more frequently from rural areas, and a higher proportion of control-group parents had higher education, although the latter difference did not reach statistical significance. Rural residence and parental educational level may influence food availability, dietary patterns, socioeconomic circumstances, and access to health information and healthcare. Thus, these characteristics represent potential confounders of the observed difference in dietary zinc intake and should be considered when interpreting the between-group comparison.

Despite the lower dietary zinc intake, serum zinc concentrations did not differ significantly between children with JIA and healthy controls. This finding is consistent with studies that have not demonstrated significantly lower circulating zinc concentrations in children with JIA despite reduced dietary intake (16, 18), although other studies have reported lower serum zinc concentrations in JIA (14, 17). Differences among studies may reflect variation in disease activity, JIA category distribution, treatment exposure, nutritional characteristics, sample size, laboratory methods, and the timing and conditions of blood sampling.

The discrepancy between dietary intake and serum zinc concentrations is biologically plausible because serum zinc represents only a small proportion of total body zinc and is tightly regulated. Circulating zinc concentrations are influenced not only by dietary intake but also by physiological factors such as age, growth, body composition, fasting status, diurnal variation, serum albumin concentration, and systemic inflammation (8, 13, 19). Inflammatory processes can alter zinc distribution through cytokine-mediated changes in hepatic zinc handling. In particular, interleukin-6 can increase hepatic expression of ZIP14 (SLC39A14), promoting redistribution of zinc from the circulation into hepatocytes and contributing to lower circulating zinc concentrations (8, 9, 19). In addition, serum albumin, an important circulating zinc-binding protein, decreases during the acute-phase response and may contribute to lower measured serum zinc concentrations (19). This concept is further supported by the BRINDA project, subsequent nutritional studies, and our previous study in hospitalized children with COVID-19, which demonstrated that lower serum zinc concentrations were associated with more pronounced inflammatory responses (8, 25). Thus, serum zinc should be regarded as a biochemical indicator of zinc status that is influenced by both nutritional and inflammatory factors rather than as a direct measure of dietary zinc intake.

In our study, BMI did not differ significantly between children with JIA and healthy controls, and none of the participants reported zinc-containing dietary supplementation. These findings reduce the likelihood that differences in BMI or supplemental zinc intake explain the observed between-group findings. However, the groups were not individually matched, and differences in rural residence and parental education may have contributed to differences in dietary intake and nutritional status. Moreover, serum albumin, fasting status, and the timing of blood sampling were not systematically incorporated into the analysis. Therefore, the absence of a significant difference in mean serum zinc concentrations should not be interpreted as evidence that dietary zinc status was equivalent between the groups.

Within the JIA cohort, children with serum zinc concentrations < 11.0 μmol/L had higher ESR, patient global assessment, physician global assessment, and clinical JADAS-27 scores than those with concentrations ≥11.0 μmol/L. The prevalence of active disease was also numerically higher in the lower-zinc group, although the difference was not statistically significant. Similarly, active joint count and overall JADAS-27 were higher numerically but did not reach statistical significance. These findings therefore provide evidence for an association between the predefined low serum zinc category and several individual measures of disease activity, but the evidence is less consistent when disease activity is assessed using the overall JADAS-27 or when serum zinc is analyzed continuously.

The lack of significant correlations between continuous serum zinc concentrations and JADAS-27 or clinical JADAS-27 further emphasizes the need for cautious interpretation. Serum zinc showed only weak inverse correlations with these measures, neither of which reached statistical significance. A weak inverse correlation with physician global assessment was statistically significant (ρ = −0.293, *p* = 0.046), but this isolated finding should also be interpreted cautiously given the number of exploratory correlations performed. Thus, the categorical and continuous analyses do not provide uniform evidence for a linear relationship between serum zinc concentration and overall disease activity.

Several biological mechanisms could potentially contribute to the observed association. Zinc has important roles in innate and adaptive immune function, including lymphocyte maturation, T-cell differentiation, cytokine regulation, antioxidant defense, and intracellular signaling (9–12). Inadequate zinc availability may influence immune regulation and inflammatory responses. Conversely, systemic inflammation can alter zinc distribution and circulating concentrations through the acute-phase response (8, 9, 19). The cross-sectional design of the present study does not allow determination of the direction of this relationship. Therefore, rather than assuming a bidirectional relationship, the observed association may reflect the combined influence of zinc-related biological processes and inflammation-associated redistribution of circulating zinc.

Our findings are partly consistent with previous studies. Islam et al. reported lower serum zinc concentrations in children with JIA and described an inverse association with disease duration (17). Sultana et al. reported lower serum zinc concentrations in children with active JIA, although the difference between JADAS-defined activity groups was not statistically significant (19). Other studies have similarly failed to demonstrate significant relationships between serum zinc concentrations and clinical characteristics of JIA (16, 18). These differences across studies may be related to variation in patient characteristics, JIA categories, disease activity, treatment exposure, sample size, laboratory methodology, and the handling of inflammatory and nutritional confounders. Taken together, the available evidence suggests that circulating serum zinc should not be regarded as a simple surrogate for dietary zinc intake and should be interpreted in the context of inflammatory and nutritional factors.

### Strengths and limitations

This study has several strengths. We simultaneously assessed dietary zinc intake and serum zinc concentrations in children with JIA and included a healthy control group for comparison. In addition, disease activity was assessed using JADAS-27 and its clinical component, allowing examination of associations between zinc status and several dimensions of disease activity. The study also included both categorical analyses based on the predefined serum zinc threshold and correlation analyses using serum zinc as a continuous variable.

Several limitations should be considered. First, this was a single-center, cross-sectional study with a relatively modest sample size and an unmatched case-control design. Although age, sex, and BMI did not differ significantly between groups, rural residence differed significantly and parental educational level also differed numerically. These sociodemographic characteristics may influence dietary patterns, socioeconomic circumstances, and access to healthcare and therefore represent potential sources of confounding. BMI alone also does not fully characterize nutritional status, while pubertal stage and more comprehensive measures of body composition were not systematically incorporated into the analysis. None of the participants reported zinc supplementation, reducing the likelihood that supplemental zinc intake contributed to the observed differences. Nevertheless, liver function, intestinal malabsorption, and other metabolic determinants of zinc status were not systematically evaluated, and residual confounding cannot be excluded.

Dietary zinc intake was estimated using a semi-quantitative FFQ based on reported intake during the preceding week. Although the questionnaire was pilot-tested and standard portion sizes and household measures were used, it was not formally validated against repeated 24-h dietary recalls or weighed food records. Dietary estimates may therefore be affected by recall and reporting bias, particularly in children requiring parental assistance with portion-size estimation. In addition, dietary zinc intake was analyzed as absolute daily intake rather than adjusted for total energy intake. Consequently, the analysis cannot fully distinguish differences in total food consumption from differences in dietary zinc density. Treatment-related effects on appetite, food intake, and nutritional status were also not incorporated into the primary dietary analysis and may represent additional sources of residual confounding.

Serum zinc was measured at a single time point and may not accurately reflect longer-term zinc status. The timing of blood sampling and fasting status were not standardized or systematically recorded, although both may influence circulating zinc concentrations. Serum albumin was not measured or incorporated into the analysis, despite its important role in zinc transport and its sensitivity to systemic inflammation (19). Additional indicators of zinc status or metabolism, such as metallothionein, ZIP14-related pathways, or hair zinc concentrations, were not assessed. Although children with acute infections, other chronic inflammatory or systemic diseases, and renal dysfunction were excluded, other factors affecting zinc metabolism could not be completely excluded. Finally, because of the cross-sectional design, the observed associations cannot establish whether lower serum zinc concentrations contribute to greater disease activity or are partly a consequence of inflammatory processes.

From a clinical perspective, our findings support the importance of considering dietary assessment as part of comprehensive nutritional care in children with JIA, particularly because dietary zinc intake below the recommended level was common in this population. Serum zinc concentrations alone may not reliably reflect dietary zinc intake, whereas dietary and serum assessments may provide different and potentially complementary information about zinc status. Further prospective multicenter studies with larger cohorts should incorporate standardized blood sampling conditions, serum albumin and other relevant nutritional and inflammatory markers, and validated dietary assessment methods. Such studies are needed to clarify the relationship between zinc status and disease activity and to determine whether correction of dietary zinc intake below the recommended level or targeted zinc supplementation has clinically meaningful effects on disease outcomes.

## Conclusion

Children with JIA had significantly lower dietary zinc intake than healthy controls, whereas mean serum zinc concentrations did not differ significantly between the groups. Within the JIA cohort, children with serum zinc concentrations below 11.0 μmol/L had higher ESR, patient and physician global assessment scores, and clinical JADAS-27 scores than those with concentrations ≥11.0 μmol/L. However, the difference in overall JADAS-27 did not reach statistical significance, and serum zinc concentration analyzed as a continuous variable was not significantly correlated with JADAS-27 or clinical JADAS-27. These findings suggest an association between low serum zinc concentrations and selected measures of disease activity but do not establish an independent or causal relationship between serum zinc status and systemic inflammation.

Dietary zinc intake and serum zinc concentrations may provide different and potentially complementary information when assessing zinc status in children with JIA. Assessment of dietary zinc intake may therefore complement clinical and laboratory evaluation, particularly given the high prevalence of intake below the age- and sex-specific recommended levels observed in this study. Further prospective studies are needed to clarify the temporal relationship between dietary zinc intake, serum zinc concentrations, inflammation, and disease activity in children with JIA and to determine whether nutritional interventions have clinically relevant effects.

## Data Availability

The raw data supporting the conclusions of this article will be made available by the authors, without undue reservation.
